# Sexual dimorphism of osteoclast reliance on mitochondrial oxidation of energy substrates in the mouse

**DOI:** 10.1172/jci.insight.174293

**Published:** 2023-12-22

**Authors:** Chao Song, Arianna Valeri, Fangfang Song, Xing Ji, Xueyang Liao, Tyler Marmo, Rebecca Seeley, Jared Rutter, Fanxin Long

**Affiliations:** 1 Translational Research Program in Pediatric Orthopaedics, Department of Surgery, Children’s Hospital of Philadelphia, Philadelphia, Pennsylvania, USA.; 2 Department of Orthopedic Surgery, Tongji Hospital, Huazhong University of Science and Technology, Wuhan, China.; 3 Department of Biochemistry, School of Medicine, University of Utah, Salt Lake City, Utah, USA.; 4 Howard Hughes Medical Institute, Chevy Chase, Maryland, USA.; 5 Department of Orthopaedic Surgery, University of Pennsylvania, Philadelphia, Pennsylvania, USA.

**Keywords:** Bone Biology, Bioenergetics, Osteoclast/osteoblast biology

## Abstract

Osteoclasts specialize in bone resorption and are critical for bone remodeling. Previous studies have shown that osteoclasts possess abundant mitochondria and derive most energy through oxidative phosphorylation (OXPHOS). However, the energy substrates fueling OXPHOS in osteoclasts remain to be fully defined. Here, we showed that osteoclast differentiation was coupled with increased oxidation of glucose, glutamine, and oleate. Transcriptomic analyses with RNA sequencing revealed marked upregulation of genes participating in OXPHOS and mitochondrial fatty acid oxidation, during osteoclast differentiation. Increased mitochondrial oxidation of long-chain fatty acids was required for osteoclast differentiation in vitro. However, blocking fatty acid oxidation in vivo, by deletion of carnitine palmitoyltransferase 1a (Cpt1a) in osteoclast progenitors, impaired osteoclast formation only in the female mice. The Cpt1a-deficient females were further protected from osteoclast activation by a high-fat diet. The males, on the contrary, exhibited normal bone resorption despite Cpt1a deletion, regardless of the dietary fat content. Moreover, concurrent deletion of mitochondrial pyruvate carrier 1 and Cpt1a, blocking mitochondrial oxidation of both glucose and fatty acids in the osteoclast lineage, failed to impede bone resorption in the males. The study therefore uncovers a female-specific dependence on mitochondrial oxidation of fatty acids and glucose in osteoclasts in vivo.

## Introduction

Osteoclasts are multinucleated cells that resorb bone to initiate bone remodeling that is essential for bone health. Osteoclasts form resorption compartments on the bone surface, locally secreting acids as well as proteolytic enzymes to dissolve minerals and digest bone matrix (1). Under healthy conditions, bone resorption is balanced by de novo bone formation to maintain bone mass, but loss of the balance in favor of resorption causes bone loss and increases bone fragility. Osteoclasts are derived from myeloid lineage cells in response to monocyte/macrophage colony-stimulating factor (M-CSF) and receptor activation of NF-κB ligand (RANKL). Whereas M-CSF promotes proliferation and survival of osteoclast precursors, RANKL drives the differentiation of osteoclasts via the induction of key transcription factors such as c-fos and nuclear factor of activated T cells 1 (Nfatc1) (2). Therapies targeting osteoclasts, including bisphosphates and the more recent RANKL-neutralizing antibody denosumab, have been the mainstay of osteoporosis treatment, but adverse effects including atypical fractures and osteonecrosis of the jaw have been reported (3). Thus, there remains a clinical need for safe and effective antiresorptive agents.

Although much is known about the signals and transcription factors regulating osteoclast differentiation, the bioenergetics of this process is just beginning to be understood. An early study with electron microscopy on bone sections documented that osteoclasts were uniquely abundant with mitochondria, compared with osteoblasts, osteocytes, or endosteal lining cells (4). More recent studies have shown that RANKL upregulates the Pparg coactivator Ppargc1b to stimulate mitochondrial biogenesis in macrophage progenitors and that genetic deletion of Ppargc1b impairs osteoclast function but not differentiation (5, 6). Knockout of Ndufs4, a subunit of complex I of the mitochondrial electron transport chain (ETC), disrupts osteoclast formation and function in the mouse (7). Moreover, the mitochondrial transcription factor Tfam is required for sustaining intracellular ATP levels in mature osteoclasts to support osteoclast survival (8). Despite the prominent role of mitochondria in osteoclasts, much remains unclear about the energy substrates fueling mitochondrial respiration in those cells.

Studies to date have documented the role of glucose in osteoclast differentiation and function. Osteoclasts from chickens, mice, and humans have been shown to rely on glucose for their resorptive activity (9–12). Glycolysis, together with oxidative phosphorylation (OXPHOS), increases with osteoclast differentiation from bone marrow macrophages (13, 14). Knockdown of lactate dehydrogenase, or deletion of the main glucose transporter Glut1, in macrophage progenitors suppresses osteoclast differentiation in vitro (13, 14). However, Glut1 knockout in myeloid lineage cells only modestly decreases the osteoclast number in vivo, and the effect is limited to female mice (13). Thus, other energy substrates besides glucose likely contribute to bioenergetics in osteoclasts.

Free fatty acids are a major class of energy substrates for mammalian cells. In particular, long-chain fatty acids (C11–20) including oleic acid, palmitic acid, and linoleic acid are the most abundant in the blood and tissues (15). Prior to β-oxidation in the mitochondria, long-chain fatty acids are transported into the matrix by a carnitine shuttle. The transport requires coordinated activities of carnitine palmitoyltransferase 1 (Cpt1) (encoded by Cpt1a, Cpt1b, or Cpt1c), which converts fatty acyl-CoA to acylcarnitine at the outer membrane of mitochondria, and Cpt2, which reverses acylcarnitine to acyl-CoA at the inner membrane (16). A previous study showed that suppression of Cpt1a in macrophages mediated polysaccharide-induced inflammatory osteolysis, but a cell-autonomous role for Cpt1a in osteoclast differentiation was not determined (17). In addition, Cpt1a upregulation was shown to promote fusion among osteoclast progenitors isolated from patients with rheumatoid arthritis, but it was unclear whether such a role was unique to the pathological condition (18). Elucidating the role of fatty acid oxidation in osteoclasts is likely to shed light on the mechanistic link between obesity and impaired bone health (19–22). In mice, high-fat diet has been shown to cause bone loss via promotion of osteoclast formation (23). Although obesity has been linked with increased fatty acid oxidation in skeletal muscle and immune cells, a potential connection in osteoclasts is yet to be explored (24, 25).

Here, by employing metabolic and genetic techniques, we show that mitochondrial fatty acid oxidation supports osteoclast differentiation both in vitro and in the mouse. Remarkably, the requirement in vivo applies only to the female mice. Osteoclasts in male mice, on the other hand, are refractory to the blockage of fatty acid or glucose oxidation or both. The study therefore uncovers unexpected sexual dimorphism of energy substrate dependence in osteoclasts in vivo.

## Results

### Fatty acid oxidation is upregulated with osteoclast differentiation.

**Figure 1:**
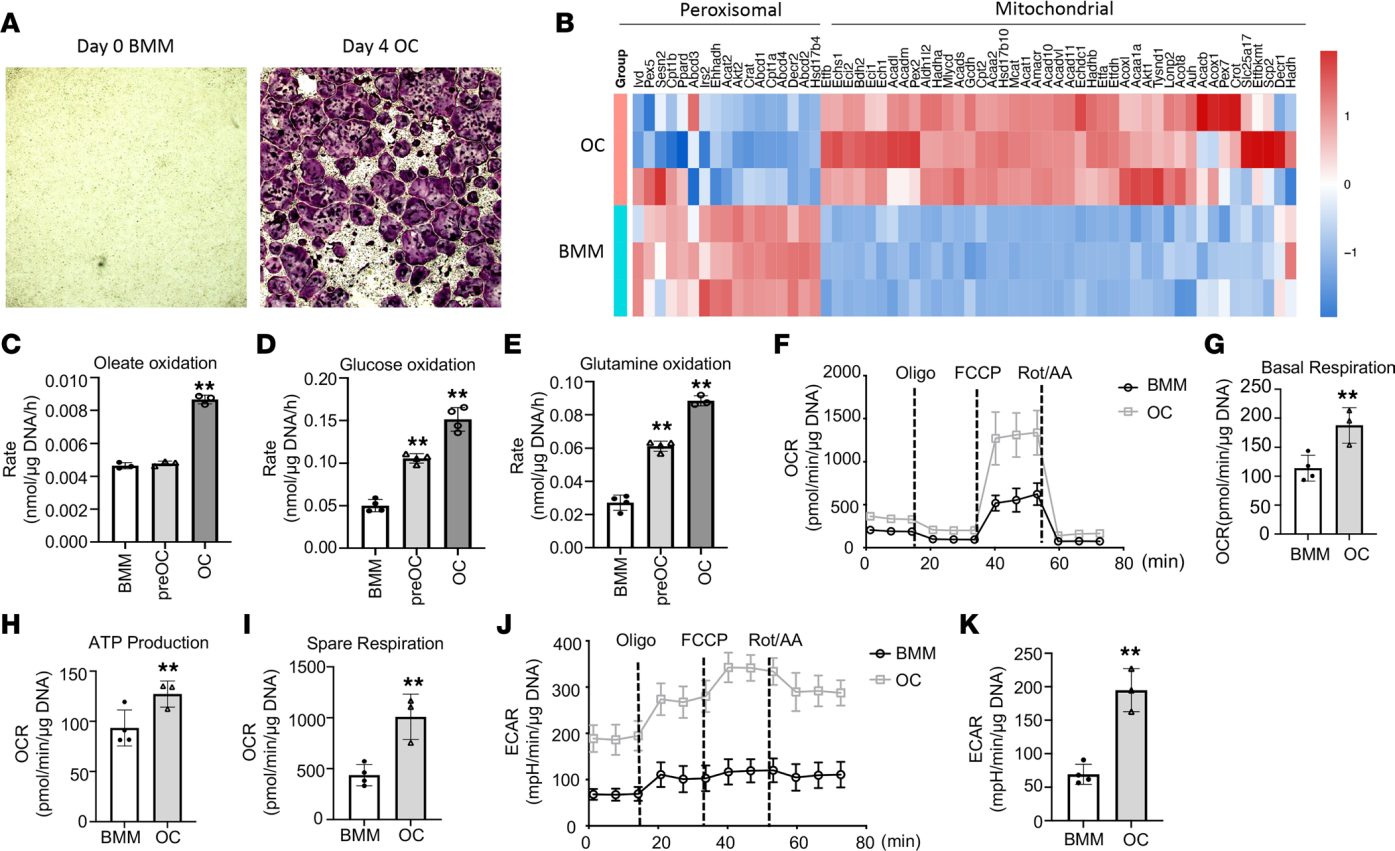
Fatty acid oxidation increases during osteoclast differentiation. (**A**) Representative images of TRAP staining for osteoclasts before and after 4 days of differentiation. Original magnification, ×40. (**B**) Heatmap for relative expression of peroxisomal or mitochondrial fatty acid β-oxidation genes in osteoclasts (OC) versus bone marrow macrophages (BMM). (**C**–**E**) Oxidation rates of various energy substrates. preOC, day 2 differentiation; OC, day 4 differentiation. **P < 0.01, 1-way ANOVA with Tukey’s multiple comparisons test. (**F**–**K**) Seahorse assays for OCR (**F**–**I**) and ECAR (**J** and **K**). **P < 0.01, 2-tailed Student’s *t* test for 2-group comparisons. Error bars: SD.

For osteoclast differentiation, bone marrow macrophages (BMMs) flushed from murine long bones were expanded in the presence of M-CSF before being induced with M-CSF and RANKL (Supplemental Figure 1A; supplemental material available online with this article; https://doi.org/10.1172/jci.insight.174293DS1). Tartrate acid-resistant phosphatase (TRAP) staining detected numerous multinucleated osteoclasts after 4 days of differentiation (Figure 1A). Efficient osteoclast differentiation was verified by the robust induction of osteoclast marker genes from day 0 through day 2 and day 4 differentiation (Supplemental Figure 1, B–G). To profile the overall transcriptomic changes, we performed RNA-Seq with BMMs before and after osteoclast differentiation (day 0 versus day 4). Ingenuity Pathway Analysis (QIAGEN) of the RNA-Seq data showed that TCA cycle and OXPHOS genes were most significantly upregulated whereas oleate biosynthesis was significantly downregulated in osteoclasts compared with BMMs (Supplemental Figure 1H). Examination of individual genes showed that those encoding the TCA enzymes or components of the ETC were all induced in osteoclasts compared with BMMs (Supplemental Figure 1, I and J). Moreover, genes related to fatty acid oxidation in either peroxisomes or mitochondria were oppositely regulated during osteoclast differentiation. Whereas those in peroxisomes, including Ehhadh, Abcd1–4, Decr2, and Hsd17b4, were downregulated, the mitochondrial fatty acid oxidation genes such as Echs1, Acads, Acadm, and Acadl were increased in osteoclasts (Figure 1B). Thus, osteoclast differentiation is coupled with marked upregulation of genes associated with mitochondrial respiration and β-oxidation of fatty acids.

To assess fatty acid oxidation directly, we quantified the CO_2_ production rate from ^14^C-labeled oleate, used as a surrogate for long-chain fatty acids. Oleate is physiologically relevant as it is among the most abundant fatty acids in the circulation and tissues of humans or mice (26). As a comparison, CO_2_ release from ^14^C-glucose or ^14^C-glutamine was also examined. Oleate oxidation did not change after 2 days of differentiation but markedly increased after 4 days (Figure 1C). On the other hand, glucose and glutamine oxidation progressively increased in both preosteoclasts and osteoclasts over BMMs (Figure 1, D and E). Thus, increased oxidation of long-chain fatty acids is selectively associated with mature osteoclast formation.

The increase in substrate oxidation predicts a greater rate of mitochondrial respiration following osteoclast differentiation. In our previous studies, preosteoclasts, upon dissociation and reseeding, displayed a higher oxygen consumption rate (OCR) than BMMs according to Seahorse measurements, but mature osteoclasts were not assessed here because of technical difficulties in dissociating them from the culture plates (13). Here, we optimized the protocol to induce osteoclast differentiation directly in the Seahorse plates before measurements without reseeding. TRAP staining verified robust osteoclast differentiation after 4 days in Seahorse wells (Supplemental Figure 1K). Compared with BMMs, mature osteoclasts markedly upregulated basal OCR, ATP production OCR, as well as spare OCR (Figure 1, F–I). In addition, osteoclasts exhibited a much higher extracellular acidification rate (ECAR) than BMMs (Figure 1, J and K). Thus, osteoclast differentiation upregulates both glycolysis and OXPHOS.

### Oleate oxidation in mitochondria promotes osteoclast formation.

**Figure 2:**
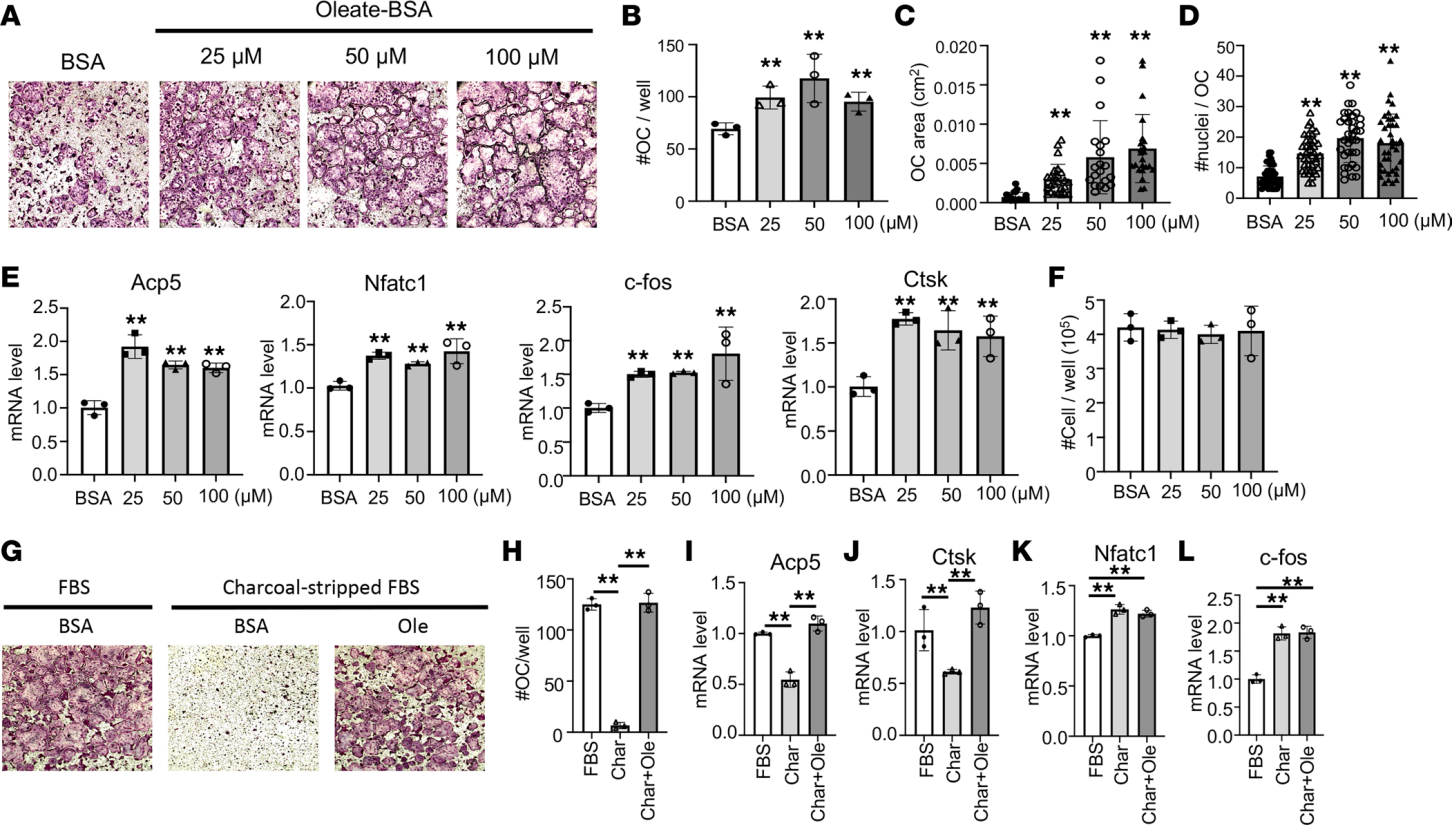
Oleate promotes osteoclast differentiation. (**A**) Representative images of TRAP-stained osteoclasts following differentiation with BSA alone or BSA-conjugated oleate at indicated concentrations. (**B**–**D**) Quantifications of the total number (**B**), area (**C**), and nucleus numbers (**D**) of mature osteoclasts (with 3 or more nuclei), following differentiation with BSA alone or BSA-conjugated oleate at indicated concentrations. (**E**) Reverse transcription quantitative PCR (RT-qPCR) analyses of osteoclast marker gene expression. (**F**) BMM cell numbers in culture with different concentrations of BSA-conjugated oleate. (**G** and **H**) Representative images (**G**) and quantification (**H**) of osteoclasts in cultures with or without charcoal-stripped FBS or oleate (Ole). Original magnification, ×40 (**A** and **G**). (**I**–**L**) RT-qPCR analyses of osteoclast marker gene expression. **P < 0.01, comparison against BSA control unless otherwise indicated as in **H**–**L**, 1-way ANOVA with Tukey’s multiple comparisons test. Error bars: SD.

We next examined the role of fatty acid oxidation in osteoclast differentiation. Addition of BSA-conjugated oleate as low as 25 μM to the differentiation media increased both the number and size of osteoclasts (Figure 2, A–D). Molecular analyses verified the upregulation of all common osteoclast markers including Acp5 (encoding TRAP), Nfatc1, c-fos, and cathepsin K (Ctsk) by oleate (Figure 2E). Addition of oleate to the growth media did not alter the cell number of BMMs after 48 hours, indicating that the increased differentiation was unlikely due to effects on cell proliferation (Figure 2F). Furthermore, when charcoal-stripped FBS (cFBS) devoid of lipid was used in lieu of regular FBS in the differentiation media, essentially no osteoclasts were formed, but differentiation was fully restored when BSA-conjugated oleate was added to the cFBS (Figure 2, G and H). Molecular analyses revealed that with cFBS the mature osteoclast markers Acp5 and Ctsk were suppressed, but they were fully restored by the added oleate (Figure 2, I and J). Interestingly, cFBS also stimulated the expression of early osteoclast markers Nfatc1 and c-fos, but that effect was not reversed by the addition of oleate, indicating that certain yet-unknown factors in FBS normally limit the expression of those genes (Figure 2, K and L). Overall, the results support a specific requirement for exogenous fatty acids during the late phase of osteoclast differentiation.

**Figure 3:**
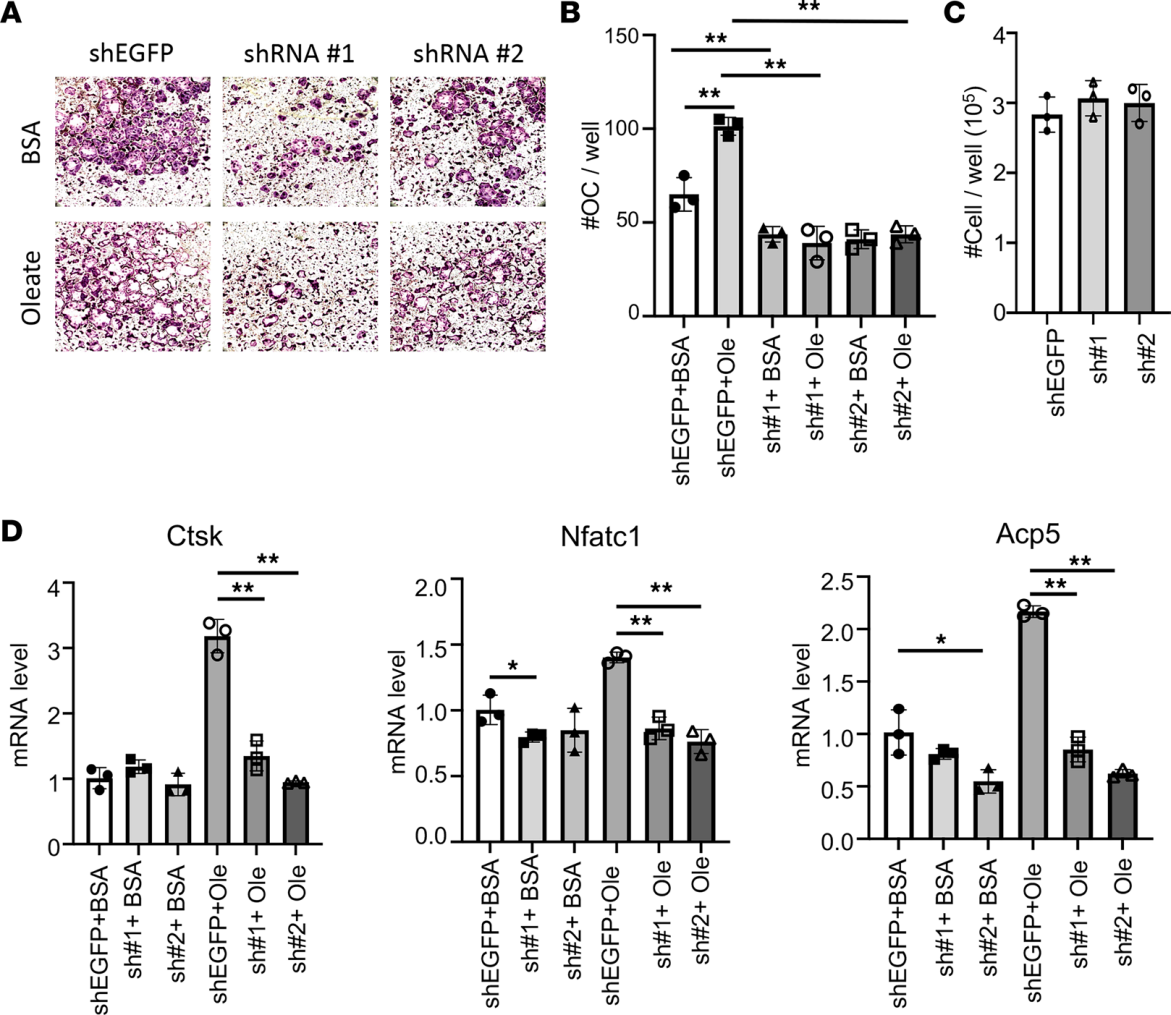
Mitochondrial fatty acid oxidation mediates oleate effect on osteoclastogenesis. (**A**) Representative images of osteoclasts in cultures with or without shRNA knockdown of Cpt2 (shRNA#1 or #2), with shEGFP as a negative control. Osteoclast differentiation was conducted with or without BSA-conjugated oleate with BSA as control. Original magnification, ×40. (**B**) Quantification of osteoclasts (with 3 or more nuclei). (**C**) BMM cell numbers with or without Cpt2 knockdown. (**D**) RT-qPCR analyses of osteoclast marker genes. *P < 0.05, **P < 0.01, 2-way ANOVA with Tukey’s multiple comparisons test. Error bars: SD.

We next sought to verify that fatty acids support osteoclastogenesis by fueling mitochondrial respiration. Cpt2 is essential for long-chain fatty acid oxidation in the mitochondria as it catalyzes the conversion of acylcarnitine to acyl-CoA at the inner mitochondrial membrane. Knockdown of Cpt2 with shRNA not only potently suppressed osteoclast differentiation under regular culture conditions but also eliminated the pro-osteoclastogenic effect of oleate (Figure 3, A and B). The requirement for Cpt2 was specific to osteoclast differentiation as the knockdown did not affect the cell number of BMMs in the growth media (Figure 3C). Molecular analyses by RT-qPCR showed that the induction of Ctsk, Nfatc1, and Acp5 by oleate was abrogated by Cpt2 shRNA (Figure 3D). Thus, mitochondrial β-oxidation of long-chain fatty acids is necessary for osteoclast differentiation in vitro.

### Deletion of Cpt1a reduces bone resorption in female mice.

**Figure 4:**
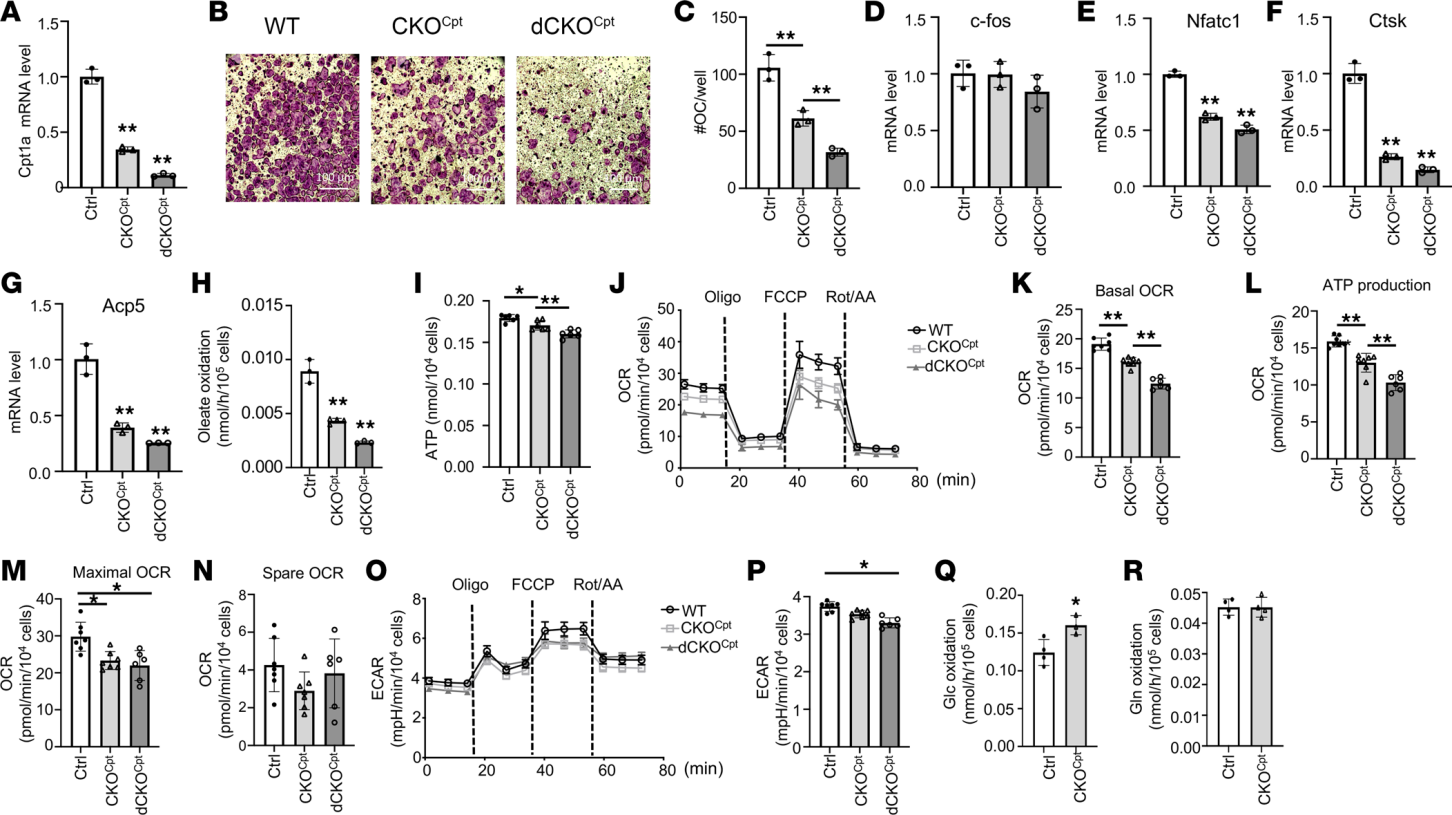
Mitochondrial fatty acid oxidation supports BMM bioenergetics and osteoclastogenesis. (**A**) RT-qPCR analyses of *Cpt1a* mRNA in BMMs from mice of different genotypes. Ctrl: Cpt1a^fl/fl^, CKO^Cpt^: LysM^Cre/+^ Cpt1a^fl/fl^, dCKO^Cpt^: LysM^Cre/Cre^ Cpt1a^fl/fl^. (**B** and **C**) Representative images (**B**) and quantification (**C**) of osteoclast differentiation in vitro. Scale bar: 100 μm. (**D**–**G**) RT-qPCR analyses of osteoclast differentiation markers following in vitro differentiation. (**H**) Oleate oxidation rate of BMMs with different genotypes. (**I**) Intracellular ATP levels in BMMs. (**J**–**N**) Seahorse measurements of OCR. (**O** and **P**) Seahorse measurements of ECAR. (**Q** and **R**) Glucose (**Q**) and glutamine (**R**) oxidation rates in BMMs. *P < 0.05, **P < 0.01, 1-way ANOVA with Tukey’s multiple comparisons test for data with 3 groups, 2-tailed Student’s *t* test for data with 2 groups. Error bars: SD.

We next examined the role of long-chain fatty acid oxidation in osteoclastogenesis by deleting Cpt1a in the mouse. RNA-Seq showed that Cpt1a was expressed at the highest level among the 3 Cpt1 genes in both BMMs and osteoclasts (Supplemental Table 2). We chose the LysM-Cre knockout/knockin allele for the deletion as it targeted the myeloid cell lineage including osteoclast progenitors. Previous studies have shown that homozygosity for the allele does not cause any bone phenotype by itself but excises the floxed genes more efficiently (27). We therefore generated Cpt1a-deficient mice harboring either 1 (LysM^Cre/+^ Cpt1a^fl/fl^) or 2 copies (LysM^Cre/Cre^ Cpt1a^fl/fl^) of the LysM-Cre allele, hereafter termed CKO^Cpt^ or dCKO^Cpt^, respectively. Expression studies verified that *Cpt1a* mRNA in BMMs was reduced by 65% and 90% of the normal level in CKO^Cpt^ and dCKO^Cpt^, respectively, irrespective of the sex of the mouse (Figure 4A). Importantly, osteoclast differentiation, with or without oleate supplementation in culture media, was impaired in both mutant cells but more severely in dCKO^Cpt^ BMMs from either male or female mice (Figure 4, B and C). Molecular analyses showed that all markers for osteoclast differentiation except for the earliest transcription factor, c-fos, were reduced in Cpt1a-deficient cells (Figure 4, D–G). The results therefore further support an important role for mitochondrial oxidation of fatty acids in osteoclast differentiation in vitro.

To verify the metabolic effect caused by Cpt1a deletion, we performed quantitative studies in BMM cultures. Oleate oxidation rate was reduced by 50% in CKO^Cpt^ versus control BMMs and further decreased by 50% in dCKO^Cpt^ cells (Figure 4H). When cultured with oleate added to media, the CKO^Cpt^ and dCKO^Cpt^ BMMs exhibited progressively lower levels of intracellular ATP than normal (Figure 4I). Seahorse extracellular flux assays detected a decrease in both basal and ATP production OCR in CKO^Cpt^ and further reduction in dCKO^Cpt^ when compared with the control BMMs (Figure 4, J–L). Both CKO^Cpt^ and dCKO^Cpt^ similarly reduced the maximal OCR but did not affect the spare OCR (Figure 4, M and N). The mutant BMMs also exhibited lower ECAR than normal, but only the dCKO^Cpt^ cells reached statistical significance (Figure 4, O and P). The reduced ECAR likely reflected an increase in pyruvate entering into the TCA cycle, resulting in reduced lactate production. Consistent with this notion, the CKO^Cpt^ BMMs exhibited a higher rate of CO_2_ production from glucose than normal whereas glutamine oxidation was not affected (Figure 4, Q and R). Thus, deletion of Cpt1a disrupts bioenergetics in osteoclast progenitors.

**Figure 5:**
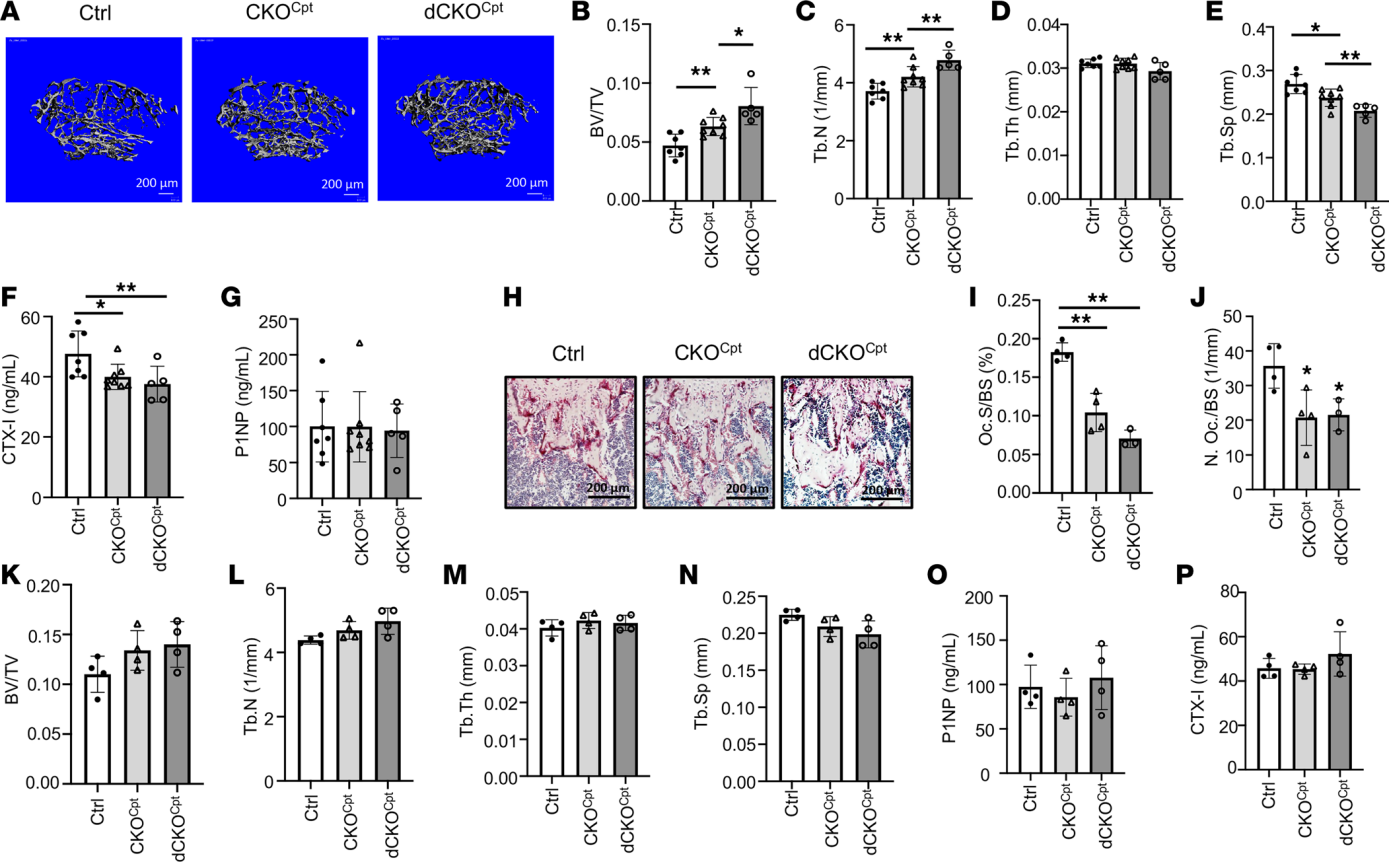
Deletion of Cpt1a suppresses osteoclast formation and increases bone mass in female mice only. (**A**) Representative μCT images of reconstructed trabecular bone of distal femurs. (**B**–**E**) Quantification of trabecular bone parameters by μCT. (**F** and **G**) Serum CTX-I and P1NP levels. (**H**) Representative TRAP staining images of distal femoral sections. Scale bar: 200 μm. (**I** and **J**) Quantification of osteoclast surface (**I**) and number (**J**) on distal femoral sections. (**K**–**N**) Quantification of trabecular bone parameters by μCT in male mice with indicated genotypes. (**O** and **P**) Serum CTX-I and P1NP levels of male mice with different genotypes. *P < 0.05, **P < 0.01, against Ctrl unless otherwise indicated, 1-way ANOVA with Tukey’s multiple comparisons test. Error bars: SD.

We next examined the potential effect of Cpt1a deletion on bone in vivo. Among the female mice, μCT analyses detected a significant increase in the trabecular bone fraction (bone volume/trabecular volume, BV/TV) of CKO^Cpt^ compared with the control and a further increase in dCKO^Cpt^ (Figure 5, A and B). The cortical bone parameters, on the other hand, were not altered in either of the mutant mice (Supplemental Figure 2, A–E). The increase in trabecular bone mass was accounted for by the increased trabecular number (Tb.N) and reduced trabecular spacing (Tb.Sp) without obvious changes in trabecular thickness (Tb.Th) (Figure 5, C–E). Bone resorption as indicated by serum C-terminal telopeptide of type I collagen (CTX-I) levels was reduced from control in both CKO^Cpt^ and dCKO^Cpt^ mice, whereas the levels of the bone formation marker procollagen type I N-terminal propeptide (P1NP) were similar across the genotypes (Figure 5, F and G). Similarly, histomorphometric analyses following TRAP staining showed that the osteoclast surface fraction (Oc.S/BS) was more severely reduced in dCKO^Cpt^ but that the osteoclast number per bone surface area (N. Oc./BS) was similarly reduced in both CKO^Cpt^ and dCKO^Cpt^ compared with the control (Figure 5, H–J). Contrary to the females, the mutant males with either 1 or 2 copies of LysM-Cre did not show any defect in either trabecular or cortical bone parameters (Figure 5, K–N, and Supplemental Figure 2, F–I). Serum biochemical assays detected no abnormalities with either CTX-I or P1NP levels in the mutant males (Figure 5, O and P). Thus, deletion of Cpt1a impairs osteoclastogenesis and increases trabecular bone mass specifically in the female mice.

### Blocking mitochondrial fatty acid oxidation eliminates HFD-induced bone loss in female mice.

**Figure 6:**
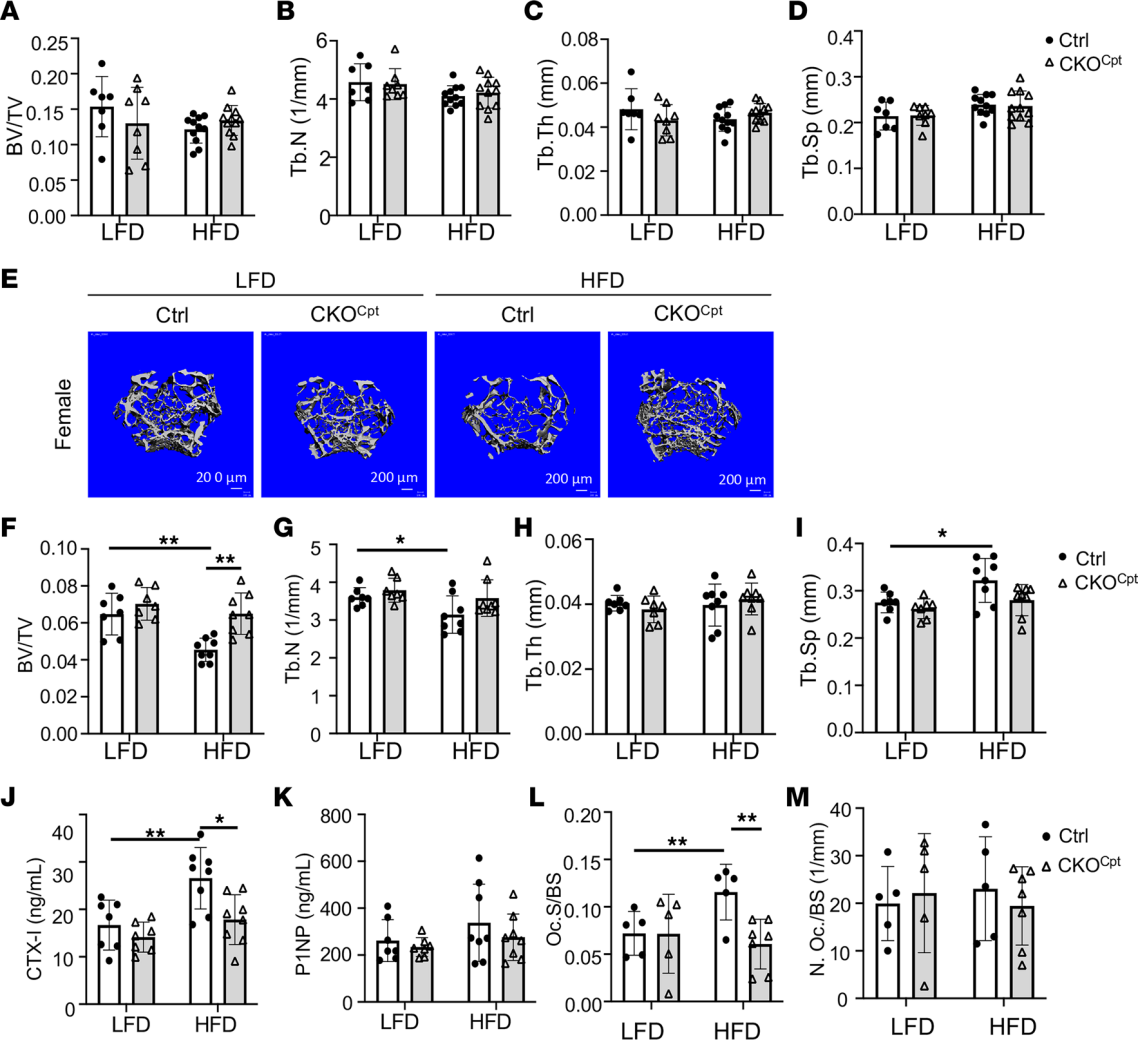
Cpt1a mediates bone loss caused by HFD in female mice only. (**A**–**D**) Quantification of trabecular bone parameters by μCT. (**E**) Representative μCT 3D images of reconstructed trabecular bone of distal femurs. (**F**–**I**) Quantification of trabecular bone parameters by μCT. (**J** and **K**) Serum levels of CTX-I (**J**) and P1NP (**K**). (**L** and **M**) Quantification of osteoclast surface (**L**) and numbers (**M**) on TRAP-stained bone sections. *P < 0.05, **P < 0.01, 2-way ANOVA with Tukey’s multiple comparisons test. Error bars: SD.

As previous studies have shown that high-fat diet (HFD) activates bone resorption in mice, we next investigated the potential role of mitochondrial fatty acid β-oxidation in mediating the catabolic effect. To this end, we subjected both control and CKO^Cpt^ mice to an HFD or a corresponding low-fat diet (LFD) for 8 weeks starting from weaning at 3 weeks of age. The 2 diets were strictly matched nutritionally except for fat content (10% versus 60% kcal) and the corresponding carbohydrate content. The male mice, like those fed the regular chow diet, did not exhibit any difference in trabecular bone between the 2 genotypes, regardless of LFD or HFD (Figure 6, A–D). Moreover, HFD did not alter the trabecular bone parameters in the control males. Thus, mitochondrial fatty acid oxidation in osteoclasts appears to be dispensable for trabecular bone homeostasis in the male mice irrespective of the fat content in the diet.

In contrast with the males, the female mice, depending on their genotypes, responded differently to HFD. The control mice exhibited a marked reduction in trabecular bone fraction (BV/TV) in response to HFD, but the CKO^Cpt^ mice showed no such change (Figure 6, E and F). Similarly, HFD decreased trabecular number and increased trabecular spacing only in the control but not the CKO^Cpt^ females (Figure 6, G–I). Unexpectedly, the CKO^Cpt^ females on LFD, unlike those fed the regular chow (containing 25% kcal in fat) as shown earlier, did not exhibit any bone defect when compared to the controls (Figure 6, E–I). Therefore, it appeared that the reliance of osteoclast bioenergetics on fatty acid oxidation was influenced by fat content in the diet. When the dietary fat supply was minimal as in the LFD (10% kcal), the contribution of fatty acid oxidation was either negligible or easily compensated by the other energy sources when Cpt1a was deleted. When the control mice were fed HFD, the serum CTX-I levels were significantly elevated, but the effect was eliminated in the CKO^Cpt^ females (Figure 6J). On the other hand, HFD did not affect P1NP levels in mice with either genotype (Figure 6K). TRAP staining on femur sections revealed that HFD increased osteoclast surface areas without altering osteoclast numbers in the control mice but had no effect in the CKO^Cpt^ mice (Figure 6, L and M). Thus, HFD promotes bone resorption in female mice mainly by increasing mitochondrial oxidation of fatty acids in osteoclasts.

### Mitochondrial oxidation of fatty acids and glucose is dispensable in osteoclasts of male mice.

**Figure 7:**
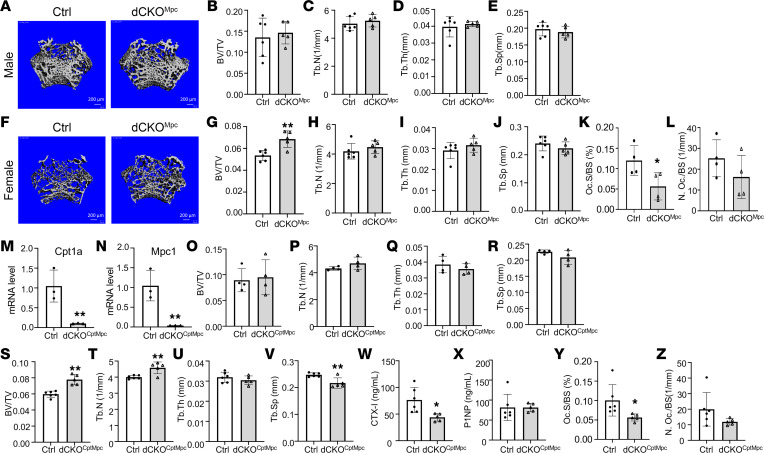
Deletion of Mpc1 alone or both Mpc1 and Cpt1a suppresses osteoclasts in female mice only. (**A**–**J**) Representative images and quantification of trabecular bone parameters by μCT in males (**A**–**E**) and females (**F**–**J**) with Mpc1 single deletion in the osteoclast lineage. dCKO^Mpc^: LysM^Cre/Cre^ Mpc1^fl/fl^. (**K** and **L**) Histomorphometric quantification of osteoclasts. (**M** and **N**) mRNA levels in BMMs as assayed by RT-qPCR. dCKO^CptMpc^: LysM^Cre/Cre^ Cpt1a^fl/fl^ Mpc1^fl/fl^. (**O**–**V**) Quantification of trabecular bone parameters by μCT in males (**O**–**R**) and females (**S**–**V**) with Mpc1 and Cpt1a double deletion. (**W** and **X**) Serum CTX-I (**W**) and P1NP (**X**) levels of female mice with double deletion. (**Y** and **Z**) Histomorphometric analyses of osteoclasts. *P < 0.05, **P < 0.01, 2-tailed Student’s *t* test. Error bars: SD.

The lack of bone phenotype in the male mice with Cpt1a deletion prompted us to examine the contribution of glucose oxidation in the mitochondria to osteoclast differentiation. To this end, we deleted mitochondrial pyruvate carrier 1 (Mpc1), which is required for the glycolytic product pyruvate to enter the mitochondria for oxidation. Here again the male mutant mice harboring 2 alleles of LysM-Cre (LysM^Cre/Cre^ Mpc1^fl/fl^) (termed dCKO^Mpc^) did not exhibit any significant change in the fractional bone mass or any other trabecular bone parameters (Figure 7, A–E). The female mutants, on the other hand, showed an overall increase in the trabecular bone mass, even though the individual contribution of trabecular thickness, number, or spacing was not statistically significant (Figure 7, F–J). Histomorphometry showed that the osteoclast fractional surface but not the relative number was significantly reduced in the Mpc1-mutant females (Figure 7, K and L). Thus, like fatty acids, mitochondrial oxidation of pyruvate, derived from glucose or other sources, appears to be dispensable for bone mass homeostasis in male mice.

Potential compensation between fatty acid and pyruvate oxidation could mask their individual contribution to osteoclast bioenergetics in the male mice. To investigate this possibility, we generated double-deletion mice with 2 alleles of LysM-Cre (LysM^Cre/Cre^ Cpt1a^fl/fl^ Mpc1^fl/fl^), designated herein dCKO^CptMpc^. Quantification by RT-qPCR verified that both Cpt1a and Mpc1 were deleted with more than 90% efficiency in BMMs in either sex (Figure 7, M and N). However, no changes were observed with the bone parameters in the male mutant mice (Figure 7, O–R). In contrast, the female mutant mice showed a clear increase in the fractional bone mass, attributable to increased trabecular number and reduced trabecular spacing (Figure 7, S–V). The excess bone mass was accounted for by a nearly 50% decrease in bone resorption as indicated by the serum CTX-I level, without any change in the bone formation marker P1NP (Figure 7, W and X). Histomorphometry verified that the fractional surface of osteoclasts was significantly reduced in the mutant female mice, whereas the relative osteoclast number showed a trend of reduction without reaching statistical significance (Figure 7, Y and Z). The results therefore underscore the remarkable resilience of osteoclasts in the male mice when mitochondrial oxidation of both fatty acids and pyruvate is blocked.

## Discussion

We have examined in detail the role of mitochondrial fatty acid oxidation in osteoclast formation in vitro and in the mouse. In keeping with the broad upregulation of genes associated with mitochondrial respiration and fatty acid oxidation, mature osteoclasts in vitro markedly increased the oxidation of long-chain fatty acids compared with their progenitors. Genetic disruption of mitochondrial fatty acid oxidation in the osteoclast lineage suppressed bone resorption in female mice fed with either regular chow or an HFD. A similar effect, however, was not observed in the males, even when both fatty acids and pyruvate oxidation in the mitochondria were blocked in osteoclasts. The study therefore not only identifies an important role for fatty acid oxidation in osteoclast formation but also reveals unexpected sexual dimorphism in energy substrate dependence of osteoclasts in vivo.

The sex-dependent effect in vivo following Cpt1a or Mpc1 deletion or both in osteoclasts warrants further investigation. Our finding is consistent with the recent report that deletion of Cpt2 in osteoclasts also specifically affects female mice (28). Our in vitro studies show that osteoclast differentiation from BMMs of either sex is equally impaired by the loss of Cpt1a or Cpt2. Thus, the female-specific effect is unlikely due to cell-intrinsic differences caused by the sex chromosomes. Previously, we have found that restricting glucose metabolism in osteoclasts by deletion of Glut1 impairs bone resorption also only in female mice (13). Collectively, the data indicate that sex-specific endocrine or paracrine factors likely play a major role in controlling energy substrate utilization by osteoclasts in vivo. Future experiments are necessary to determine whether the male hormones confer a greater level of plasticity in metabolic switching to either alternative substrates or pathways in osteoclasts.

The in vitro studies have revealed stage-specific preferences for different energy substrates during osteoclast differentiation. The increase in fatty acid oxidation occurred only in the large multinucleated osteoclasts, which formed after 4 days of differentiation, but not in the preosteoclasts formed after 2 days in our culture system. In contrast, oxidation of glucose and glutamine oxidation increased in both preosteoclasts and osteoclasts compared with BMMs. The surge in fatty acid oxidation coincides with the fusion of mononuclear preosteoclasts to form multinuclear osteoclasts, but the underlying mechanism is unclear at present. Apart from the obvious contribution to energy production, increased fatty acid oxidation could alter the levels of certain metabolites to influence either cell membrane dynamics or the epigenetic regulation in support of mature osteoclast formation.

Our finding about the male mice fed with HFD is at odds with a previous study that used the same LFD and HFD as ours but detected an increase in bone resorption in the male mice (23). One obvious difference was in the starting age for LFD or HFD feeding, namely 5 weeks in the previous study versus 3 weeks here. The different starting age could be significant as the bone resorption rate can vary considerably with age. Furthermore, the regular chow (LabDiet 5015) that our mice consumed before switching to matched HFD/LFD was different from that used in the previous study (LabDiet 5010), with ours containing a significantly higher amount of fat (25% versus 12.7% kcal). It is possible that the different fat contents consumed by the mice before switching to the HFD led to different responses. We did not monitor whole-body metabolism in our studies, but the previous study reported notable hyperglycemia and metabolic dysfunction in their mice, which could have separately contributed to the reported bone resorption defect, independent of fatty acid fueling of osteoclasts. Overall, the different outcomes highlight the highly variable metabolic responses by osteoclasts to different diets, and further studies are needed for a mechanistic understanding.

## Methods

### Mice.

Cpt1a^fl/fl^ (strain 032778) and LysM-Cre (strain 004781) mice have been previously described and were purchased from The Jackson Laboratory (29, 30). The Mpc1^fl/fl^ mice are as previously described (31). The mice were fed regular chow diet (LabDiet 5015) unless otherwise indicated. Certain mice were fed an HFD (D12492, Research Diets Inc.) or an LFD (D12450B, Research Diets Inc.) in a head-to-head comparison.

### Osteoclast differentiation.

BMMs were isolated and cultured as previously described (32). Briefly, both ends of the femur and the tibia were removed before the bone marrow was flushed out with cell culture media. Bone marrow cells were cultured for 3 days on Petri dishes in growth media; the growth media were reconstituted from a custom-made MEMα powder (Gibco) with addition of fresh glucose, pyruvate, and glutamine, supplemented with 10% FBS (Atlanta Biologicals) together with 10% CMG14-12 cell culture supernatant. The CMG14-12 supernatant containing M-CSF was prepared as previously described (33). The adherent BMMs were then dissociated with 0.25% Trypsin-EDTA (Gibco) and reseeded with growth media in 96- or 24-well plates at the density of 7.5 × 10^4^ cells/cm^2^. On the following day, the growth media were replaced with differentiation media, i.e., reconstituted custom-made MEMα containing 10% FBS, 1:50 CMG14-12 supernatant, and 75 ng/mL recombinant RANKL (PeproTech). Osteoclasts were detected with TRAP staining (MilliporeSigma).

### Metabolic assays.

For Seahorse assays, 2 × 10^4^ BMMs were seeded in a Seahorse plate and incubated in growth or differentiation media for 2 or 4 days, with media replaced every 12 hours. Osteoclast differentiation in Seahorse plates was verified with TRAP staining on day 4. Before Seahorse measurements, the cell culture media were replaced with Seahorse XF DMEM supplemented with 5.5 mM glucose, 2 mM glutamine, 1 mM pyruvate, and 100 mM BSA-conjugated oleate and then placed in a CO_2_-free incubator for 1 hour. For Mito Stress tests, 2 μM oligomycin, 3 μM FCCP, and 1 μM rotenone/antimycin A were used. The Seahorse data were normalized to genomic DNA as measured in lysed cells with BioTek Cytation 5 (Agilent Technologies) following staining with Hoechst 33342 (Thermo Fisher Scientific).

For steady-state ATP measurements, BMMs were seeded in 96-well plates at 7.5 × 10^4^ cells/cm^2^ and cultured in growth media overnight. A total of 100 μL fresh media per well was replaced 4 hours before intracellular ATP levels were measured with the CellTiter-Glo kit (Promega). The ATP levels were normalized to the cell numbers.

Substrate oxidation assays were conducted as previously described (32). In brief, ^14^C-labeled oleate, glucose, or glutamine was mixed as tracers with the bulk unlabeled substrates. Full media containing the different tracers were incubated with cells for 4 hours before an aliquot of the media was transferred to a 1.5 mL Eppendorf tube containing 200 μL perchloric acid. Released CO_2_ was captured by a filter paper fitted inside the tube cap and saturated with sodium hydroxide. The radiation counts on the filter papers were used to compute the substrate oxidation rates.

### RNA-Seq and RT-qPCR.

Total RNA was extracted with RNeasy kits (QIAGEN) before being subjected to high-throughput RNA-Seq (GENEWIZ) or RT-qPCR. For RNA-Seq, RNA samples were quantified using Qubit 2.0 Fluorometer (Life Technologies), and RNA integrity was checked using Agilent TapeStation 4200. The RNA-Seq libraries were prepared using the NEBNext Ultra II RNA Library Prep Kit for Illumina following manufacturer’s instructions (New England Biolabs). The sequencing libraries were clustered on 1 flowcell lane. After clustering, the flowcell was loaded on the Illumina HiSeq instrument (4000 or equivalent) according to manufacturer’s instructions. The samples were sequenced using a 2 × 150 bp paired-end configuration. Image analysis and base calling were conducted by the Control software. Raw sequence data (.bcl files) generated from the sequencer were converted into fastq files and demultiplexed using Illumina’s bcl2fastq 2.17 software. After investigation of the quality of the raw data, sequence reads were trimmed to remove possible adapter sequences and nucleotides with poor quality using Trimmomatic v.0.36. The trimmed reads were mapped to the reference genome available on ENSEMBL using the STAR aligner v.2.5.2b. After extraction of gene hit counts, the gene hit counts table was used for downstream differential expression analysis. Using DESeq2, a comparison of gene expression between the groups of samples was performed. The Wald test was used to generate *P* values and log_2_ fold-changes.

For RT-qPCR, cDNA was produced with High Capacity RNA-to-cDNA kit (Thermo Fisher Scientific) and then used for quantitative PCR with SYBR green PCR Master Mix (Applied Biosystems) in a QuantStudio 3 machine (Applied Biosystems). Gene-specific quantitative PCR primers are listed in Supplemental Table 1.

### Oleate-BSA conjugation.

To make 4 mM BSA, 5.5 g BSA was mixed with 11.5 mL Dulbecco’s phosphate-buffered saline followed by gentle shaking at room temperature for 3 hours. To conjugate oleate with BSA, 24.36 mg of sodium oleate was added to 2 mL of H_2_O in a water bath at 70°C for approximately 20 minutes before mixing quickly with 6 mL of the 4 mM BSA stock solution prewarmed to 70°C.

### Bone histology and μCT analyses.

Femurs were fixed in 10% formalin for 48 hours at room temperature and then decalcified in 14% EDTA solution (pH 7.2) for 14 days with the solution changed every 4 days. The bones were then processed, embedded in paraffin, and sectioned at 6 μm thickness. The sections were subjected to TRAP staining for osteoclast detection. Osteoclasts were quantified with BIOQUANT OSTEO 2021 V21.5.60.

For μCT analyses, femurs fixed with 10% formalin were scanned using μCT 45 (Scanco Medical AG) at 4.5 μm isotropic voxel size according to the guidelines of American Society of Bone and Mineral Research (34). For trabecular bone analysis, 200 slices starting at 70 slices under the growth plate of the distal femur were analyzed with the threshold set at 330. For cortical bone analysis, 35 slices before and after the middle slice of the femur diaphysis were analyzed with the threshold set at 370.

### Serum biochemical measurements.

Mice were fasted for 6 hours before blood collection via retro-orbital bleeding. CTX-I and P1NP levels were measured with RatLaps ELISA and Rat/Mouse P1NP EIA Kit, respectively (Immunodiagnostic Systems, Ltd.).

### Statistics.

For data with 2 groups, statistical tests were 2-tailed unpaired Student’s *t* tests. For data with multiple groups, statistical tests were 1-way or 2-way ANOVA with Tukey’s multiple comparisons test, as indicated in figure legends. *P* < 0.05 was considered statistically significant.

### Study approval.

All mouse work was approved by the Children’s Hospital of Philadelphia Institutional Animal Care and Use Committee (IACUC approval IAC 21-001296).

### Data availability.

Raw data from RNA-Seq experiments are available through NCBI GEO depository with accession number GSE235773.

## Author contributions

CS and FL were responsible for designing research studies; CS, AV, FS, XJ, XL, TM, and RS were responsible for conducting experiments and acquiring data; CS and FL were responsible for analyzing data and writing the manuscript; and JR was responsible for providing reagents.

## Supplementary Material

Supplemental data

Supporting data values

## Version 1 11/02/2023

In-Press Preview

## Version 2 12/22/2023

Electronic publication
