# Supplementary material for: Sexual dimorphism of osteoclast reliance on mitochondrial oxidation of energy substrates in the mouse
Source: JCI Insight. 2023 Dec 22;8(24):e174293. doi: 10.1172/jci.insight.174293 (PMC10807709; doi:10.1172/jci.insight.174293)
Supplement: Supplemental data [file jciinsight-8-174293-s103.pdf]

Supplemental Table I. Nucleotide sequences of primers used in RT-qPCR

**Gene**	Forward primer	Reverse primer
Acp5	AAGCGCAAACGGTAGTAAGG	CGTCTCTGCACAGATTGCAT
Nfatc1	GGTAACTCTGTCTTTCTAACCTTAAGCTC	GTGATGACCCCAGCATGCACCAGTCACA
c-fos	CAAGCGGAGACAGATCAACTTG	TTTCCTTCTCTTTCAGCAGATTGG
Ctsk	AGGCAGCTAAATGCAGAGGGTACA	AGCTTGCATCGATGGACACAGAGA
Dcstamp	ACAAACAGTTCCAAAGCTTGC	TCCTTGGGTTCCTTGCTTC
Atp6v0d2	GAAGCTGTCAACATTGCAGA	TCACCGTGATCCTTGCAGAAT
Cpt1a	GGTCTTCTCGGGTCGAAAGC	TCCTCCCACCAGTCACTCAC

Supplemental Table II. Relative mRNA levels of Cpt1 genes during osteoclast differentiation

Gene Name	Day 0-1	Day 0-2	Day 0-3	Day 4-1	Day 4-2	Day 4-3
Cpt1a	110.3279	104.8234	103.0636	69.8292	52.3812	63.9754
Cpt1b	0.3882	0.3777	0.3329	0.4516	0.1421	0.2744
Cpt1c	0.4003	0.4003	0.6634	2.1488	1.6245	1.6731

Shown are TPM by RNA-seq in three independent samples at day 0 or 4 of differentiation.

Supplemental Figures

Figure S1. Osteoclast differentiation from BMM in vitro. (A) A schematic of bone marrow macrophages (BMM) preparation and osteoclast differentiation. (B-G) Expression of osteoclast marker genes assessed by RT-qPCR following different days of differentiation. (H) Most significantly affected pathways in osteoclasts (OC) vs BMM as detected by IPA analysis of RNA-seq data. Positive Z scores indicate increased expression in osteoclasts. (I, J) Heatmap of TCA (I) and ETC (J) genes based on RNA-seq of OC versus BMM. (K) Representative images for osteoclast differentiation in Seahorse plates. Purple staining indicates TRAP-positive preosteoclasts (day 2) or osteoclasts (day 4). *p<0.05, **p<0.01, against day 0, one-way ANOVA with Tukey's multiple comparisons test. Error bars: SD.

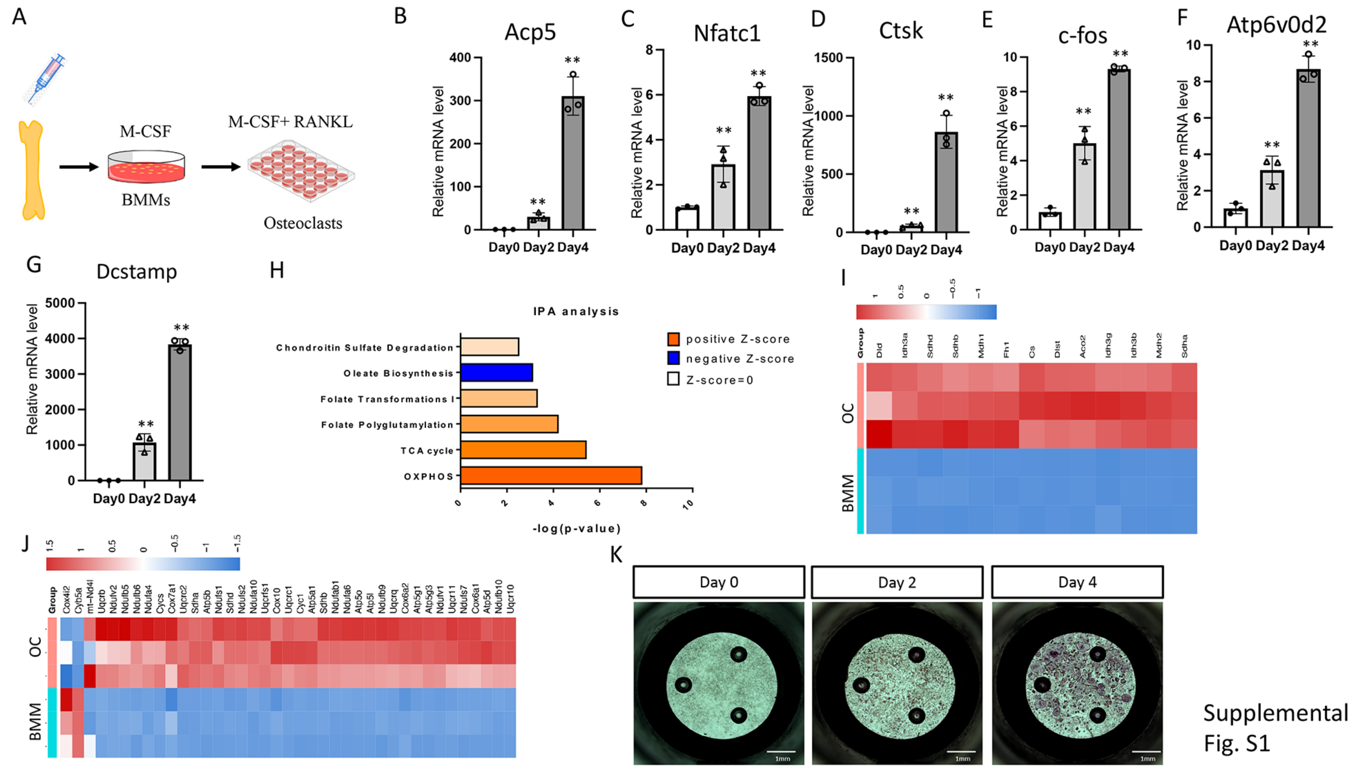

Figure S2. Deletion of Cpt1a does not affect cortical bone in either sex. (A-E) Representative cortical bone images and cortical bone parameters of μCT in female mice. (F-I) Quantification of cortical bone parameters by μCT in male mice. No statistical significance detected between mutant and wild-type mice by one-way ANOVA with Tukey's multiple comparisons test. Error bars: SD.

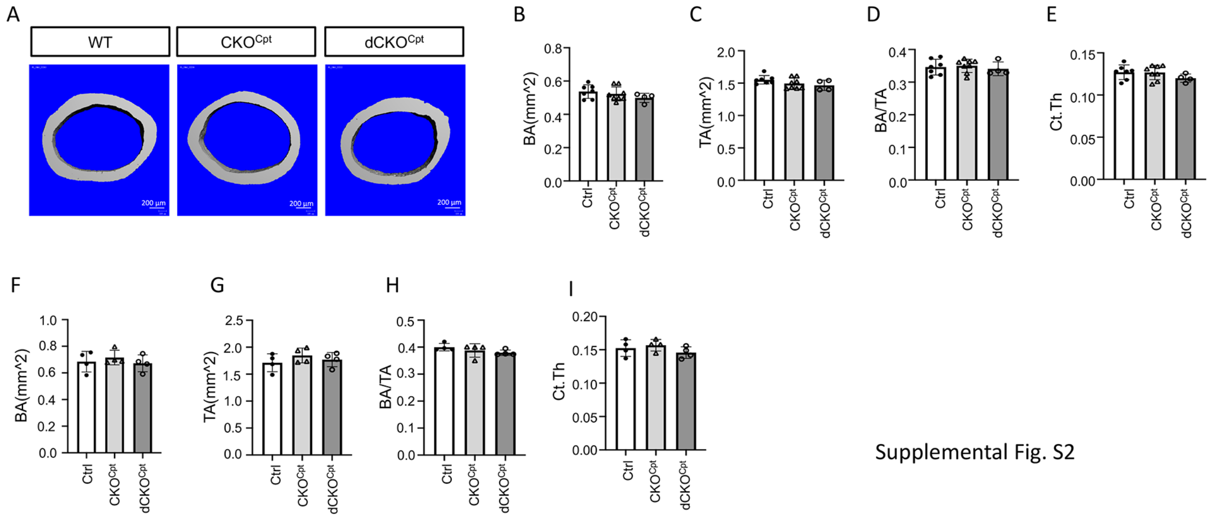

Supplemental Fig. S2
